# The genetic relationship between handedness and neurodevelopmental disorders^☆^

**DOI:** 10.1016/j.molmed.2013.10.008

**Published:** 2014-02

**Authors:** William M. Brandler, Silvia Paracchini

**Affiliations:** 1 MRC Functional Genomics Unit, Department of Physiology, Anatomy, and Genetics, University of Oxford, Oxford, OX1 3PT, UK; 2 Wellcome Trust Centre for Human Genetics, University of Oxford, Oxford, OX3 7BN, UK; 3 School of Medicine, University of St Andrews, St Andrews, KY16 9TF, UK

**Keywords:** cerebral asymmetry, ciliogenesis, corpus callosum, dyslexia, handedness, schizophrenia

## Abstract

Handedness and brain asymmetry have been linked to neurodevelopmental disorders such as dyslexia and schizophrenia. The genetic nature of this correlation is not understood. Recent discoveries have shown handedness is determined in part by the biological pathways that establish left/right (LR) body asymmetry during development. Cilia play a key role in this process, and candidate genes for dyslexia have also been recently shown to be involved in cilia formation. Defective cilia result not only in LR body asymmetry phenotypes but also brain midline phenotypes such as an absent corpus callosum. These findings suggest that the mechanisms for establishing LR asymmetry in the body are reused for brain midline development, which in turn influences traits such as handedness and reading ability.

## Highlights

• Genes controlling left/right (LR) body asymmetry also influence handedness.
• Some genes associated with handedness or dyslexia are expressed in cilia.
• Cilia defects lead to both LR body asymmetry and brain midline phenotypes.
• Cilia may play a role in brain midline development, handedness, and dyslexia.

## Linking left-handedness and cerebral asymmetry with human disorders

Worldwide, more than 85% of individuals are right-handed [1,2]. This suggests there is an advantage to being right-handed, but also begs the question of why there are left-handers. Researchers have hypothesized that instead of being part of normal variation, there is a disadvantage to being left-handed. Consequently, left-handedness has been linked to all types of disorders, such as alcoholism [3], allergies and autoimmune disorders [4], autism [5], and these are only the disorders beginning with the letter ‘a’.

Because hand-writing preference is easy to measure, being a simple tick-box on a questionnaire, it is often included in clinical or epidemiological studies, but results are typically only published if they are significant. Accordingly, many associations between handedness and disorders or traits appear to be due to publication bias, where initial small studies have shown associations that have not been replicated in larger follow-up studies or meta-analyses (Box 1). The only systematic review of the relationship between handedness and developmental disorders was performed in 1990 and found no evidence to suggest there are any associations [6]. However, a meta-analysis of 3175 individuals with schizophrenia has shown that it is associated with an increased prevalence of left-handedness (odds ratio = 1.81 [7]), but mixed results have also been reported [8].

Being right-handed implies left-hemisphere dominance (see Glossary) for fine motor control, and handedness correlates with brain hemispheric asymmetries [9]. Furthermore, there is a weak correlation between language lateralization and handedness; 96% of strong right-handers, as compared with 73% of strong left-handers, show left-hemisphere dominance for language [10]. However, the classical model of language centers in Broca's and Wernicke's areas of the left hemisphere is too simplistic. Language processing involves a complex network of regions distributed throughout the brain [11]. There is growing support from neuroimaging studies that atypical or weak cerebral lateralization is associated with neurodevelopmental disorders such as specific language impairment and dyslexia [12]. Similarly, magnetic resonance imaging studies have suggested that the planum temporale is less asymmetric in individuals with schizophrenia [13–15]. Although making connections is tempting, it remains difficult to determine cause and effect. Does weak cerebral laterality cause the disorder or vice versa, or do genetic influences underlie both weak laterality and neurodevelopmental disorders (pleiotropy) [12]?

Understanding the molecular basis of these traits may contribute to answering these questions. This review will chart recent developments in the fields of genetics and genomics that are beginning to offer insights into the relationship between handedness, cerebral asymmetry, and neurodevelopmental disorders, with a particular focus on schizophrenia and dyslexia.

## The genetic architecture of handedness: nongenetic, monogenic, or polygenic?

Laland argues that humans have a universal predisposition towards right-handedness that derives from a series of selective sweeps throughout evolution [16]. His theoretical model suggests that our genes favor right-handedness, and any variation between individuals derives purely from environmental influences, such as cultural pressure to conform [17]. Conversely, single gene models that can explain the observed variation in hand preferences have been proposed [18–20].

A study of over 25 000 twin pairs has shown that the preferred hand for writing or drawing is a weak genetic trait with a heritability of 24% [21], which appears to rule out exclusively nongenetic arguments. However, even though single gene theories fit data on the prevalence of handedness, linkage studies have failed to identify a single locus, pointing instead to different regions of the genome, including 2p12–q11 [22,23], 10q26 [24], 12q21–23 [25], and Xq21 [26]. Furthermore case/control genome-wide association studies (GWASs) for handedness have found no statistically significant associations, despite adequate sample sizes to detect a single locus with a strong effect size [27,28].

Taking these studies in combination, McManus *et al.* concluded that handedness cannot be controlled by a single genetic locus. Instead, they estimated that at least 40 loci underlie the variation in this trait [29]. Given the universality of right-handedness among humans [1], it seems that an innate bias towards being right-handed has been selected for during evolution as Laland suggests [16]. However, this bias is probably influenced by both cultural and environmental pressures as well as genetic variants, as expected for a polygenic trait.

## Shared genetics between handedness and schizophrenia

The proposed link between schizophrenia and left-handedness [7] has led to numerous molecular investigations of its relationship to handedness. Linkage studies have pointed to regions on chromosome 2p carrying genetic factors implicated in the development of both schizophrenia [30,31] and handedness [22,23]. One study selected four candidate genes within the overlapping region and genotyped common single nucleotide polymorphisms (SNPs), which resulted in finding a haplotype associated with relative hand skill in a set of 222 dyslexic siblings (assessed by the peg-board task; Box 2) upstream of leucine-rich repeat transmembrane neuronal protein 1 (*LRRTM1*) when paternally inherited [32]. Although this finding does not replicate in independent cohorts unaffected with dyslexia, the same haplotype was also associated with schizophrenia when paternally inherited [32,33].

The *LRRTM1* finding suggests that schizophrenia and left-handedness may have overlapping genetic susceptibility factors; it is therefore possible that the same variants that modulate risk for schizophrenia are also associated with handedness. Testing of 16 variants across different genes that have been associated with schizophrenia in a cohort of 444 healthy individuals did not support this hypothesis, finding no associations with handedness or footedness [34]. These susceptibility variants for schizophrenia only have a small effect on risk for developing the disorder, and possibly have an even smaller effect on risk for left-handedness. It is therefore improbable that any one single variant will be strongly associated enough with handedness to be consistently detected in small cohorts.

## *PCSK6*: a molecular link between handedness and dyslexia

**Figure 1:**
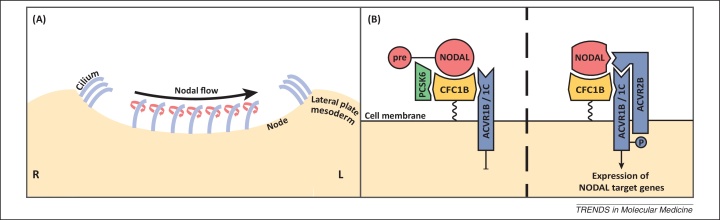
Establishment of left/right (LR) asymmetry during development. **(A)** Cross-section of the developing embryo during gastrulation viewed from the posterior. The node is a pit that forms transiently at the midline during gastrulation and contains two types of primary cilia (blue lines). Posteriorly angled clockwise rotating cilia create a leftward flow which is detected by mechanosensory cilia [51,53], and transduced to an increase of intracellular calcium ions in the left side triggering asymmetrical expression of genes such as NODAL [52]. **(B)** Zoomed in representation of NODAL signaling at the surface of a cell on the left side of both the node and lateral plate mesoderm. Cryptic family protein 1B (CFC1B) is tethered to the membrane by a glycosylphosphatidylinositol (GPI; a glycolipid) anchor [94], and it recruits NODAL proprotein (pre-NODAL), proprotein convertase subtilisin/kexin type 6 (PCSK6), and activin type I receptors (ACVR1B/ACVR1C) [40–42,95]. PCSK6 then cleaves pre-NODAL into an active form, and a type II activin receptor (ACVR2B) forms a complex with the NODAL ligand, type I receptors, and CFC1B [42]. Type I, type II receptors, and NODAL exist as homodimers and the binding of the NODAL ligand causes the receptors to combine into a heterotetrameric complex (for simplicity proteins are shown as monomers) [96]. Phosphorylation of type I receptors by ACVR2B then transmits the NODAL signal via a signal transduction pathway that activates expression of NODAL target genes, specifying that the cell is on the left side of the embryo [97]. Variants in both PCSK6 and ACVR2B have been associated with relative hand skill in individuals with dyslexia [37].

The language-related nature of dyslexia has also prompted investigations for a possible association with handedness. A GWAS for relative hand skill, using the peg-board task, has been performed in two cohorts, one consisting of individuals with dyslexia (*n* = 728), and a general population cohort unaffected with dyslexia (*n* = 2666). Individuals with dyslexia are slower overall at performing the peg-board task compared with controls but there is no difference in the distribution of their relative hand skills (PegQ) [35–37]. One statistically significant SNP associated with relative hand skill was reported in individuals with dyslexia, which is located in an intron of proprotein convertase subtilisin/kexin type 6 (*PCSK6*; Table 1) [37,38]. PCSK6 is a protease that cleaves NODAL into an active form [39] when anchored to the cell surface by cryptic family protein 1B (CFC1B) (Figure 1) [40,41]. NODAL then signals through type I and type II activin receptors (such as ACVR1B/ACVR1C [42] and ACVR2B [43]) to trigger the development of left/right (LR) asymmetry [44] (Figure 1). This pathway is conserved across bilaterians from snails to vertebrates [45,46]. *Pcsk6* knockout mice display asymmetry defects such as heterotaxia, which is an abnormal distribution of body organs [39]. Therefore, given its role in LR asymmetry development, *PCSK6* is an extremely interesting biological candidate for handedness. However, it is curious that the PCSK6 association with PegQ appears to be specific in the dyslexia cohort [37].

## Handedness and left/right body asymmetry

The most highly associated variant with relative hand skill in the general population cohort, although not significant at a genome-wide threshold, is located in *GPC3* [37]. When *GPC3* is disrupted in mice it causes heart and lung asymmetry defects [47]. Further investigation of the GWAS data through gene set enrichment analysis (GSEA; [48]) shows an overrepresentation of other variants associated with relative hand skill located in the human orthologs of genes that also cause LR asymmetry phenotypes when knocked out in mice. Three phenotypes in particular show association both in the general population and in the dyslexia cohort: heterotaxia, situs inversus (a reversal of organ asymmetry), and double outlet right ventricle (a heart asymmetry defect). Therefore, the same biological mechanism for determining LR asymmetry in the body plays a role in the development of handedness, regardless of a dyslexia diagnosis. However, when comparing the cohort of individuals with dyslexia to the general population cohort, the associations are observed for different SNPs or genes within those same biological pathways. This suggests both allelic and locus heterogeneity between the cohorts, which could be explained by epistasis between genes involved in dyslexia and those involved in handedness. In addition, an independent study found that a variable number tandem repeat (VNTR), in proximity to the genome-wide significant associated SNP in *PCSK6*, is associated with degree of handedness (i.e., extreme left or right handedness versus mixed handedness) in a general population cohort not selected for dyslexia, further supporting this hypothesis [49].

## Cilia, handedness, and dyslexia

**Figure 2:**
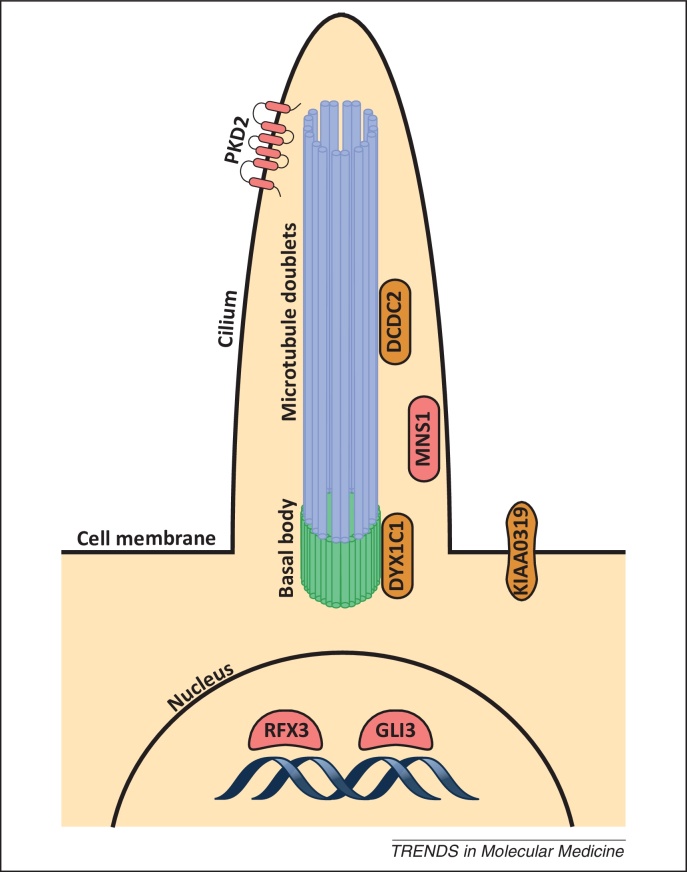
Cilia and the biology of handedness and dyslexia. Subcellular localization of genes associated with either relative hand skill (pink) or dyslexia (orange) are highlighted. Regulatory factor X 3 (RFX3) is a transcription factor important for ciliogenesis, regulating assembly, growth, and beating efficiency of cilia [98]. GLI family zinc finger 3 (GLI3) is also a transcription factor expressed at primary cilia [99], and its expression is regulated by RFX3 [64]. The unidirectional fluid flow created by cilia rotation that breaks asymmetry is detected via the Ca^2+^ channel polycystic kidney disease 2 (PKD2), on the membrane of mechanosensory cilia [100]. Meiosis-specific nuclear structural protein 1 (MNS1) localizes to cilia, and mice in which the gene is disrupted display severe left/right (L/R) asymmetry defects [56]. Candidate genes for dyslexia are also expressed in cilia [71]. Kazusa Institute AA0319 (KIAA0319) is a transmembrane protein [101]. Dyslexia susceptibility 1 candidate gene 1 (DYX1C1) localizes at the basal body and doublecortin domain containing 2 (DCDC2) on the microtubules; both genes regulate cilia length [72,73,75].

The biological mechanism that determines LR asymmetry in embryonic development involves the rotation of motile cilia that create a leftward flow in the node during gastrulation (Figure 1) [50]. This flow is detected by non-motile mechanosensory cilia [51]. The protein product of the polycystic kidney disease 2 (*PKD2*) gene localizes to the cilium, and is involved in transducing this signal into an increase of intracellular calcium ions, on the left side of the node, that act as a secondary messenger to trigger left-sided expression of genes such as NODAL [52]. The expression of NODAL on the left edge of the node induces further expression of itself and other genes in a positive feedback loop that spreads expression to the lateral plate mesoderm and signals left-sided positional information to cells (Figure 1) [53]. Cilia mediate many important functions in development and defective cilia cause many syndromes or disease, known as ciliopathies, which can cause asymmetry defects such as situs inversus [54,55]. Four out of the five most strongly associated genes in the GSEA of the GWAS study for relative hand skill in the dyslexia cohort are involved in ciliogenesis: meiosis-specific nuclear structural protein 1 (*MNS1*), regulatory factor X 3 (*RFX3*), GLI family zinc finger 3 (*GLI3*), as well as *PKD2* (Figure 2, Table 1) [37]. Disruption of *Mns1*, *Rfx3*, or *Pkd2* in mice causes situs inversus [56–58]. Surprisingly, individuals with situs inversus do not show an increased likelihood of being left-handed [59]; therefore, it was previously thought that mechanisms which regulate body asymmetries were distinct from those that regulate brain asymmetry [60]. Yet genes that cause situs inversus appear to be important in the development of handedness. It is possible, therefore, that compensatory mechanisms allow for the normal development of handedness in individuals with situs inversus, suggesting that the development of handedness is more complex than just involving early LR asymmetry determining genes. However, although handedness may not reverse in situs inversus, brain asymmetry as a whole can reverse. Two brain imaging studies that each included three individuals with situs inversus have shown a significant reversal of the typical pattern of right-frontal and left-occipital petalia asymmetry [61,62], of which one study also showed a significant reversal of language dominance [62]. Situs inversus is a rare disorder affecting 1/10 000 individuals [63], and large-scale studies have not been performed yet.

Ciliopathies are also known to cause two structural phenotypes in the brain: an absent corpus callosum and an absent cerebellar vermis [55]. These two midline structures connect the hemispheres of the cerebrum and cerebellum, respectively. *RFX3* and *GLI3* are known to be involved in both ciliogenesis and corpus callosum development. RFX3 regulates the expression of *Gli3* in the telencephalon in mice, which in turn regulates the distribution of guidepost neurons necessary for corpus callosum formation [64]. Mice deficient in RFX3 show an absent corpus callosum [64]; similarly, mutations in *GLI3* in humans also cause an absent corpus callosum [65]. Interestingly, *PCSK6* is also highly expressed in the corpus callosum [66]. However, the evidence for a relationship between handedness and corpus callosum size is inconclusive (reviewed in [67]), and a study of 12 infants with an absent corpus callosum show no difference in right-handedness compared with controls [68].

To date, very few candidate genes have been proposed for dyslexia susceptibility, but most seem to play a role in early stages of brain development, and neuronal migration more specifically [69]. The migration of neurons can be directed by the flow of cerebrospinal fluid, which is circulated by motile cilia [70], and dyslexia candidate genes have recently been implicated in cilia function. A cilia-related coexpression module derived from microarray datasets finds that the dyslexia associated genes, doublecortin domain containing 2 (*DCDC2*), dyslexia susceptibility 1 candidate gene 1 (*DYX1C1*), and Kazusa Institute AA0319 (*KIAA0319*) are coexpressed in cilia (Figure 2) [71]. *Dyx1c1* is upregulated during ciliogenesis and localizes to centrioles and basal bodies of cilia in multiciliated tracheal epithelial cells in mice [72]. Disrupting *Dyx1c1* in mice causes laterality defects, chronic airway disease, and male infertility, resembling primary ciliary dyskinesia (PCD) [73]. Similarly, inhibition of *dyx1c1* in zebrafish reduces the length of cilia and produces asymmetry phenotypes such as situs inversus [72]. In humans, recessive loss-of-function mutations in *DYX1C1* have been identified in 12 patients with PCD [73]. *DCDC2* has a doublecortin-like (DCX) domain involved in microtubule length regulation [74], and overexpression of *Dcdc2* increases the length of cilia in rat hippocampal neurons [75]. A striking feature of KIAA0319 is the presence of five (PKD) domains [76]. PKD2 and other PKD family members play key roles in cilia function and LR asymmetry development and lead to ciliopathies [77].

Intriguingly, individuals with an absent corpus callosum or cerebellar vermis display motor coordination problems [78,79]. Motor coordination and balance problems have been consistently observed in individuals with dyslexia, and it has been hypothesized that cerebellar dysfunction underlies both reading and coordination difficulties in dyslexia [80].

Taken together, these data and observations suggest that genes implicated in dyslexia may be involved in ciliogenesis.

## Concluding remarks and future perspectives

Recent developments have shown that handedness is controlled in part by genes that play a key role in the establishment of LR asymmetry early in development through NODAL signaling and ciliogenesis. These pathways control development of both LR asymmetry in the body and also midline structures in the brain. In parallel, it is emerging that dyslexia candidate genes play a role in ciliogenesis. We propose that the biological mechanisms for establishing LR asymmetry in the body are reused for the development of midline structures in the brain, which in turn influences traits such as handedness and reading ability. Detailed phenotyping in combination with increasingly affordable DNA genotyping and sequencing will be a powerful tool to unravel the full complexity of handedness, cerebral asymmetry, and neurodevelopmental disorders.

## Figures and Tables

**Table 1:** Genes associated with handedness related measures

Gene	Gene function	Study type^b^	Cohort size	Cohort affection status	Refs
***ACVR2B***	Receptor for NODAL	GSEA of GWAS data	728	Individuals with dyslexia	[37]
***GLI3***	Ciliogenesis	GSEA of GWAS data	728	Individuals with dyslexia	[37]
***GPC3***	Heart/lung asymmetry	Strongest association in GWAS	2666	General population	[37]
***LRRTM1***^a^	Neuronal development	Candidate gene	222	Dyslexic siblings	[32]
***MNS1***	Ciliogenesis	GSEA of GWAS data	728	Individuals with dyslexia	[37]
***PCSK6***	Cleaves NODAL into an active form	GW significant GWAS association	728	Individuals with dyslexia	[37,38]
		Candidate gene	1113	General population	[49]
***PKD2***	Detects nodal flow	GSEA of GWAS data	728	Individuals with dyslexia	[37]
***RFX3***	Ciliogenesis	GSEA of GWAS data	728	Individuals with dyslexia	[37]

a *LRRTM1* is also associated with schizophrenia [32,33].

b Abbreviations: GWAS, genome-wide association study; GW, genome wide; GSEA, gene set enrichment analysis.

## Box 1 Publication bias and the file drawer problem in handedness research

Handedness is inexpensive and easy to measure and thus is often included in studies as a ‘bonus factor’, even though it may have very little scientific merit for the study in question [81,82]. Any significant result leads to an additional publication, whereas nonsignificant results are often forgotten, gathering dust in the file drawers of researchers. This publication bias is known as the file drawer problem [83].

A recent example is a study that linked an isoform of the Alzheimer's associated gene, apolipoprotein E (*APOE*), and handedness [84]. There are two medically relevant common protein coding variants in the gene that exist in three combinations (or isoforms) – ɛ2, ɛ3, and ɛ4. Carriers of ɛ4 are more likely to develop Alzheimer's disease [85,86], carriers of ɛ2 are protected against Alzheimer's, whereas the wild type ɛ3 carriers show typical odds. ɛ2 carriers showed a higher prevalence of left-handedness (29.2%) relative to ɛ3 homozygotes (8.9%) and ɛ4 carriers (6.1%) in a cohort of 147 individuals [84].

The odds of being left-handed in ɛ2 carriers compared with noncarriers is 3.88, which is unusually large for a genetic association study of a common variant; these typically have odds ratios of 1.1–1.5 [87]. Indeed, out of 1570 published GWASs, only 55 (3.5%) report odds ratios that can better this [88], including the strong association of ɛ4 with Alzheimer's (odds ratio = 4.1 [86]).

However, a meta-analysis of 19 540 individuals across 11 different cohorts (including the original study [84]) failed to find any association between ɛ2 and left-handedness [89]. A further study of 4438 individuals also failed to report any association between *APOE* variants and either left handedness or higher left-hand grip strength [90]. This example highlights the need for replication of genetic associations, particularly where the initial study cohort is small (i.e., <500), in order to reduce publication of false positives.

## Box 2 How to measure handedness

There are three different questionnaires commonly used to assess handedness: the Edinburgh handedness inventory [91], the Annett handedness questionnaire [92], and the Crovitz–Zener score [93]. The Edinburgh handedness inventory is a 20-point questionnaire on the preferred hand (left or right) for a number of tasks from writing to threading a needle [91]. The Annett and Crovitz–Zener scores ask 12 and 14 questions, respectively, both of which have ten questions that overlap with the Edinburgh inventory [92,93]. A laterality quotient can be derived from these scores, which shows a J-shaped distribution, that is, most people use their right hand for most tasks, a sizeable minority use their left hand for most tasks, and an even spread of low numbers of individuals that lie somewhere in between.

Participants are typically subsequently categorized in terms of direction of handedness: either left-, mixed-, or right-handed, or in terms of degree of handedness: either consistently preferring one hand, or inconsistent. Because there are no standard cut-off values for these distinctions, this can potentially lead to post-hoc classifications. Simulations have shown that by varying the criteria used for classifying subjects as ‘left’ or ‘right’ handed, under certain circumstances the probability of obtaining a significant result can be as high as 40% [82].

Although most people do prefer one hand over the other, handedness is a matter of degree, not type. The peg-board task is a useful phenotype in this respect because it is quantitative, easy and quick to measure, and offers more information about dexterity and degree of handedness than a questionnaire-based assessment of preferred hand. The task measures the time taken to move a row of pegs from one location to another with the left hand (L) and right hand (R) separately [18]. This provides a measure of relative hand skill (PegQ) that is normally distributed with a mean shifted to the right, because most individuals are faster at this task with their right hand. PegQ is highly correlated with hand preference: for every one standard deviation shift to the right of the PegQ distribution, individuals are 13 times more likely to be right handed [37].

## Glossary

Activin receptor (ACVR): transmembrane receptor that transduces signals from ligands such as NODAL. Type I receptors (ACVR1B and ACVR1C) are essential for signaling, and type II receptors (e.g., ACVR2B) are essential for ligand binding.
Allele: alternate forms of the same gene generated by mutations.
Allelic heterogeneity: a trait that is influenced by different mutations in the same gene.
Cerebellar vermis: region of the cerebellum lying between and connecting the two hemispheres.
Cerebral asymmetry: anatomical, functional, or physiological differences between the left and right hemispheres of the brain. For example, the planum temporale is typically larger in the left hemisphere than in the right. It has been proposed that reduced cerebral asymmetry correlates with neurodevelopmental disorders such as schizophrenia.
Cilia: hair-like organelles that protrude from the surface of a diverse array of cell types. Classically they are defined as motile (beating or rotating) or non-motile, and perform an array of functions, for example, generating a unidirectional flow that breaks bilateral LR symmetry, sweeping mucus and dirt away from lungs, enabling sperm to swim, and sensing flow. Non-motile and rotating primary cilia are internally structured with nine microtubule doublets anchored by a centriole-derived basal body and arranged in a circular pattern called the axoneme.
Ciliogenesis: the building of cilia during development. Defects in ciliogenesis are known as ciliopathies, and they can cause LR asymmetry defects such as situs inversus.
Corpus callosum: bundle of neural fibers that connect the left and right hemispheres of the cerebrum.
Cryptic family protein 1B (Cripto/CFC1B): cell membrane bound coreceptor that binds to both NODAL and PCSK6, facilitating their interaction.
Developmental dyslexia: specific reading disability that cannot be accounted for by intelligence or lack of appropriate opportunity to learn; it affects approximately 5–10% of the population.
Doublecortin-like domain (DCX): evolutionarily conserved protein domain that binds to microtubules and typically occurring in tandem. The *DCX* gene is mutated in X-linked neuronal migration defects.
Double outlet right ventricle: developmental abnormality where both the pulmonary artery and the aorta connect to the right ventricle in the heart.
Epistasis: where an allele at one locus modifies the effects one or more alleles at another locus.
Gene set enrichment analysis (GSEA): a computational method that tests whether an *a priori* specified set of genes shows an overrepresentation of association with a particular phenotype than expected by chance.
Genome-wide association study (GWAS): a hypothesis free approach where hundreds of thousands or millions of SNPs across the genome are tested for association with a trait. There are two types: case/control and quantitative association studies. In case/control, the frequency of variants is compared in cases with the trait versus controls. In a quantitative association study, genotypes are correlated with a measure that is normally distributed.
Genome-wide significance: given the large number of independent tests in a GWAS, a stringent threshold *P*-value of ≤ 5 × 10^–8^ is required for significance. This reduces the false positive rate; however, it leads to a large number of false negatives as many SNPs are only weakly associated with traits, and therefore show more modest *P*-values (depending on the sample size).
Glypican 3 (*GPC3*): gene involved in controlling the growth of the body and organs. When disrupted in mice it causes heart and lung asymmetry defects, as well as overgrowth and various types of cancer.
Haplotype: combination of alleles at adjacent locations on a chromosome that are inherited together.
Hemisphere dominance: the brain is lateralized for specific functions, for example, hand preference and language. The dominant hemisphere is the preferred hemisphere used for performing specific tasks; however, this does not necessarily preclude the other hemisphere from playing a role.
Heterotaxia: a congenital defect that results in abnormal positioning of body organs (also known as situs ambiguous). This is contrasted with the typical positioning of organs known as situs solitus with, for example, the heart and stomach on the left and the liver on the right.
Heterotetrameric complex: four proteins of more than one type held together by noncovalent interactions.
Homodimer: two identical proteins held together by noncovalent interactions.
Intron: a noncoding sequence within a gene that is removed by RNA splicing during transcription.
Lateral plate mesoderm: during early embryonic development the embryo consists of three layers: the endoderm (inner layer), mesoderm (middle), and ectoderm (outer). The mesoderm goes on to form tissues such as muscle and red blood cells, and the lateral plate mesoderm refers to mesoderm located at the periphery of the embryo.
Leucine-rich repeat transmembrane neuronal protein 1 (*LRRTM1*): a variant in this gene has been associated with both handedness and schizophrenia, when inherited paternally. This gene is imprinted, which means that the expression of the gene depends on whether it was inherited from the mother (suppressed) or the father (expressed).
Linkage studies: the closer two genetic variants are located to each other on a chromosome, the less likely they are to be separated by recombination during meiosis. By testing segregation of known markers with a phenotype within families, it is possible to identify portions of chromosome that are likely to carry genetic variants responsible for the phenotype.
Monogenic: a trait or disorder caused by an allele in a single gene, for example, cystic fibrosis or Huntington's disease.
Morphogen: a signaling molecule that diffuses along a gradient in embryonic development. The concentration of the morphogen determines the cellular response and helps cells determine their position in the embryo.
*NODAL*: morphogen that plays a key role in determining LR asymmetry. In vertebrates, NODAL signals to cells to determine the left side thus breaking bilateral symmetry.
Node: a concave-shaped structure important for LR asymmetry determination. It is formed transiently at the midline during gastrulation.
Ortholog: gene present in two or more species that originated from a single gene present in the last common ancestor of those species.
Planum temporale: region of the cerebral cortex located on the superior temporal gyrus that is involved in language. It is usually larger in the left cerebral hemisphere.
Pleiotropy: where one gene has an effect on multiple phenotypic traits.
Polygenic: a trait or disorder whose phenotype is influenced by the combination of alleles in many genes, for example, height or IQ.
Primary ciliary dyskinesia (PCD): ciliopathy characterized by chronic airway disease, laterality defects, and male infertility.
Proprotein: an inactive protein precursor that can be turned into an active form by post-translational modifications, for example, cleaving with a protease.
Proprotein convertase subtilisin/kexin type 6 (*PCSK6*): protease that cleaves NODAL proprotein into an active form (also known as *PACE4*).
Protease: an enzyme that cleaves proteins, by hydrolyzing peptide bonds in a process known as proteolysis.
Schizophrenia: a psychiatric disorder characterized by a range of psychological symptoms, including hallucinations and delusions. It affects ∼1% of the population.
Single gene models: propose that genetic variation in handedness is under the control of alleles at a single locus in a gene, that is, it is a monogenic trait. Recent technological advances, such as GWAS, have shown that handedness cannot be controlled by a single gene.
Single nucleotide polymorphism (SNP): a position (base pair/nucleotide) in the genome that is variable between individuals of the same species.
Situs inversus: a reversal of body organ asymmetry, that is, the heart, spleen, and stomach are on the right, and the liver is on the left.
Variable number tandem repeat (VNTR): short nucleotide sequence that is repeated in tandem, with variable numbers of repeats observed across individuals.

## References

[1] Marchant L.F., McGrew W.C. (1998). Human handedness: an ethological perspective. Hum. Evol..

[2] Corballis M.C. (1991). The Lopsided Ape: Evolution of the Generative Mind.

[3] Bakan P. (1973). Left-handedness and alcoholism. Percept. Mot. Skills.

[4] Geschwind N., Behan P. (1982). Left-handedness: association with immune disease, migraine, and developmental learning disorder. Proc. Natl. Acad. Sci. U.S.A..

[5] Colby K.M., Parkison C. (1977). Handedness in autistic children. J. Autism Childh. Schizophr..

[6] Bishop D.V.M. (1990). Handedness and Developmental Disorder.

[7] Dragovic M., Hammond G. (2005). Handedness in schizophrenia: a quantitative review of evidence. Acta Psychiatr. Scand..

[8] Deep-Soboslay A. (2010). Handedness, heritability, neurocognition and brain asymmetry in schizophrenia. Brain.

[9] Geschwind D.H. (2002). Heritability of lobar brain volumes in twins supports genetic models of cerebral laterality and handedness. Proc. Natl. Acad. Sci. U.S.A..

[10] Knecht S. (2000). Handedness and hemispheric language dominance in healthy humans. Brain.

[11] Fisher S.E., Marcus G.F. (2006). The eloquent ape: genes, brains and the evolution of language. Nat. Rev. Genet..

[12] Bishop D.V. (2013). Cerebral asymmetry and language development: cause, correlate, or consequence?. Science.

[13] Shapleske J. (1999). The planum temporale: a systematic, quantitative review of its structural, functional and clinical significance. Brain Res. Brain Res. Rev..

[14] Sommer I. (2001). Handedness, language lateralisation and anatomical asymmetry in schizophrenia: meta-analysis. Br. J. Psychiatry.

[15] Clark G.M. (2010). Asymmetry loss is local rather than global in adolescent onset schizophrenia. Schizophr. Res..

[16] Laland K.N. (2008). Exploring gene–culture interactions: insights from handedness, sexual selection and niche-construction case studies. Philos. Trans. R. Soc. Lond. B: Biol. Sci..

[17] Laland K.N. (1995). A gene–culture model of human handedness. Behav. Genet..

[18] Annett M. (1985). Left, Right, Hand and Brain: The Right Shift Theory.

[19] Klar A.J.S. (1996). A single locus, RGHT, specifies preference for hand utilization in humans. Cold Spring Harb. Symp. Quant. Biol..

[20] McManus C. (2004). Right Hand, Left Hand: The Origins of Asymmetry in Brains, Bodies, Atoms and Cultures.

[21] Medland S.E. (2009). Genetic influences on handedness: data from 25,732 Australian and Dutch twin families. Neuropsychologia.

[22] Francks C. (2003). Confirmatory evidence for linkage of relative hand skill to 2p12-q11. Am. J. Hum. Genet..

[23] Francks C. (2002). A genomewide linkage screen for relative hand skill in sibling pairs. Am. J. Hum. Genet..

[24] Van Agtmael T. (2002). Parametric and non-parametric linkage analysis of several candidate regions for genes for human handedness. Eur. J. Hum. Genet..

[25] Warren D.M. (2006). Heritability and linkage analysis of hand, foot, and eye preference in Mexican Americans. Laterality.

[26] Laval S.H. (1998). Evidence for linkage to psychosis and cerebral asymmetry (relative hand skill) on the X chromosome. Am. J. Med. Genet..

[27] Eriksson N. (2010). Web-based, participant-driven studies yield novel genetic associations for common traits. PLoS Genet..

[28] Armour J.A. (2013). Genome-wide association study of handedness excludes simple genetic models. Heredity (Edinb.).

[29] McManus I.C. (2013). Multilocus genetic models of handedness closely resemble single-locus models in explaining family data and are compatible with genome-wide association studies. Ann. N. Y. Acad. Sci..

[30] DeLisi L.E. (2002). A genome-wide scan for linkage to chromosomal regions in 382 sibling pairs with schizophrenia or schizoaffective disorder. Am. J. Psychiatry.

[31] Lewis C.M. (2003). Genome scan meta-analysis of schizophrenia and bipolar disorder, part II: schizophrenia. Am. J. Hum. Genet..

[32] Francks C. (2007). LRRTM1 on chromosome 2p12 is a maternally suppressed gene that is associated paternally with handedness and schizophrenia. Mol. Psychiatry.

[33] Ludwig K.U. (2009). Supporting evidence for LRRTM1 imprinting effects in schizophrenia. Mol. Psychiatry.

[34] Ocklenburg S. (2013). Cholecystokinin A receptor (CCKAR) gene variation is associated with language lateralization. PLoS ONE.

[35] Francks C. (2003). Familial and genetic effects on motor coordination, laterality, and reading-related cognition. Am. J. Psychiatry.

[36] Stoodley C.J., Stein J.F. (2006). A processing speed deficit in dyslexic adults? Evidence from a peg-moving task. Neurosci. Lett..

[37] Brandler W.M. (2013). Common variants in left/right asymmetry genes and pathways are associated with relative hand skill. PLoS Genet..

[38] Scerri T.S. (2011). PCSK6 is associated with handedness in individuals with dyslexia. Hum. Mol. Genet..

[39] Constam D.B., Robertson E.J. (2000). SPC4/PACE4 regulates a TGFβ signaling network during axis formation. Genes Dev..

[40] Blanchet M.H. (2008). Cripto localizes Nodal at the limiting membrane of early endosomes. Sci. Signal..

[41] Blanchet M.H. (2008). Cripto recruits Furin and PACE4 and controls Nodal trafficking during proteolytic maturation. EMBO J..

[42] Reissmann E. (2001). The orphan receptor ALK7 and the Activin receptor ALK4 mediate signaling by Nodal proteins during vertebrate development. Genes Dev..

[43] Kosaki R. (1999). Left–right axis malformations associated with mutations in ACVR2B, the gene for human activin receptor type IIB. Am. J. Med. Genet..

[44] Schier A.F., Shen M.M. (2000). Nodal signalling in vertebrate development. Nature.

[45] Levin M. (2005). Left–right asymmetry in embryonic development: a comprehensive review. Mech. Dev..

[46] Grande C., Patel N.H. (2009). Nodal signalling is involved in left–right asymmetry in snails. Nature.

[47] Ng A. (2009). Loss of glypican-3 function causes growth factor-dependent defects in cardiac and coronary vascular development. Dev. Biol..

[48] Segre A.V. (2010). Common inherited variation in mitochondrial genes is not enriched for associations with type 2 diabetes or related glycemic traits. PLoS Genet..

[49] Arning L. (2013). VNTR polymorphism is associated with degree of handedness but not direction of handedness. PLoS ONE.

[50] Hirokawa N. (2012). Cilia, KIF3 molecular motor and nodal flow. Curr. Opin. Cell Biol..

[51] Tabin C.J., Vogan K.J. (2003). A two-cilia model for vertebrate left–right axis specification. Genes Dev..

[52] Takao D. (2013). Asymmetric distribution of dynamic calcium signals in the node of mouse embryo during left–right axis formation. Dev. Biol..

[53] Babu D., Roy S. (2013). Left–right asymmetry: cilia stir up new surprises in the node. Open Biol..

[54] Fliegauf M. (2007). When cilia go bad: cilia defects and ciliopathies. Nat. Rev. Mol. Cell Biol..

[55] Badano J.L. (2006). The ciliopathies: an emerging class of human genetic disorders. Annu. Rev. Genomics Hum. Genet..

[56] Zhou J. (2012). MNS1 is essential for spermiogenesis and motile ciliary functions in mice. PLoS Genet..

[57] Bonnafe E. (2004). The transcription factor RFX3 directs nodal cilium development and left–right asymmetry specification. Mol. Cell. Biol..

[58] Pennekamp P. (2002). The ion channel polycystin-2 is required for left–right axis determination in mice. Curr. Biol..

[59] McManus I.C. (2004). Handedness and situs inversus in primary ciliary dyskinesia. Proc. Biol. Sci..

[60] Sun T., Walsh C.A. (2006). Molecular approaches to brain asymmetry and handedness. Nat. Rev. Neurosci..

[61] Kennedy D.N. (1999). Structural and functional brain asymmetries in human situs inversus totalis. Neurology.

[62] Ihara A. (2010). Neuroimaging study on brain asymmetries in situs inversus totalis. J. Neurol. Sci..

[63] Torgersen J. (1950). Situs inversus, asymmetry, and twinning. Am. J. Hum. Genet..

[64] Benadiba C. (2012). The ciliogenic transcription factor RFX3 regulates early midline distribution of guidepost neurons required for corpus callosum development. PLoS Genet..

[65] Vortkamp A. (1991). GLI3 zinc-finger gene interrupted by translocations in Greig syndrome families. Nature.

[66] Johnson J.M. (2003). Genome-wide survey of human alternative pre-mRNA splicing with exon junction microarrays. Science.

[67] Corballis M.C. (2012). Right hand, left brain: genetic and evolutionary bases of cerebral asymmetries for language and manual action. WIREs Cogn. Sci..

[68] Sacco S. (2006). Agenesis of the corpus callosum and the establishment of handedness. Dev. Psychobiol..

[69] Paracchini S. (2007). The genetic lexicon of dyslexia. Annu. Rev. Genomics Hum. Genet..

[70] Sawamoto K. (2006). New neurons follow the flow of cerebrospinal fluid in the adult brain. Science.

[71] Ivliev A.E. (2012). Exploring the transcriptome of ciliated cells using in silico dissection of human tissues. PLoS ONE.

[72] Chandrasekar G. (2013). The zebrafish orthologue of the dyslexia candidate gene *DYX1C1* is essential for cilia growth and function. PLoS ONE.

[73] Tarkar A. (2013). DYX1C1 is required for axonemal dynein assembly and ciliary motility. Nat. Genet..

[74] Coquelle F.M. (2006). Common and divergent roles for members of the mouse DCX superfamily. Cell Cycle.

[75] Massinen S. (2011). Increased expression of the dyslexia candidate gene DCDC2 affects length and signaling of primary cilia in neurons. PLoS ONE.

[76] Velayos-Baeza A. (2007). Alternative splicing in the dyslexia-associated gene KIAA0319. Mamm. Genome.

[77] Marshall W.F. (2008). The cell biological basis of ciliary disease. J. Cell Biol..

[78] Economou A., Katsetos C.D. (2012). Patterns of cognitive and fine motor deficits in a case of Dandy–Walker continuum. J. Child Neurol..

[79] Mueller K.L. (2009). Bimanual motor coordination in agenesis of the corpus callosum. Behav. Neurosci..

[80] Stoodley C.J., Stein J.F. (2011). The cerebellum and dyslexia. Cortex.

[81] Fairweather H. (1976). Sex-differences in cognition. Cognition.

[82] Bishop D.V.M. (1990). How to increase your chances of obtaining a significant association between handedness and disorder. J. Clin. Exp. Neuropsychol..

[83] Rosenthal R. (1979). The file drawer problem and tolerance for null results. Psychol. Bull..

[84] Bloss C.S. (2010). APOE genotype is associated with left-handedness and visuospatial skills in children. Neurobiol. Aging.

[85] Raber J. (2004). ApoE genotype accounts for the vast majority of AD risk and AD pathology. Neurobiol. Aging.

[86] Coon K.D. (2007). A high-density whole-genome association study reveals that APOE is the major susceptibility gene for sporadic late-onset Alzheimer's disease. J. Clin. Psychiatry.

[87] Manolio T.A. (2009). Finding the missing heritability of complex diseases. Nature.

[88] Hindorff, L.A. *et al. A Catalog of Published Genome-Wide Association Studies* (www.genome.gov/gwastudies; Accessed July 2013)

[89] Piper B.J. (2013). Non-replication of an association of Apolipoprotein E2 with sinistrality. Laterality.

[90] Hubacek J.A. (2012). Lack of an association between left-handedness and APOE polymorphism in a large sample of adults: results of the Czech HAPIEE study. Laterality.

[91] Oldfield R.C. (1971). The assessment and analysis of handedness: The Edinburgh Inventory. Neuropsychologia.

[92] Annett M. (1970). A classification of hand preference by association analysis. Br. J. Psychol..

[93] Crovitz H.F., Zener K. (1962). A group-test for assessing hand- and eye-dominance. Am. J. Psychol..

[94] Watanabe K. (2007). Requirement of glycosylphosphatidylinositol anchor of Cripto-1 for trans activity as a Nodal co-receptor. J. Biol. Chem..

[95] Yeo C., Whitman M. (2001). Nodal signals to Smads through Cripto-dependent and Cripto-independent mechanisms. Mol. Cell.

[96] Yamashita H. (1994). Formation of hetero-oligomeric complexes of type I and type II receptors for transforming growth factor-β. J. Biol. Chem..

[97] Wrana J.L. (1994). Mechanism of activation of the TGF-β receptor. Nature.

[98] El Zein L. (2009). RFX3 governs growth and beating efficiency of motile cilia in mouse and controls the expression of genes involved in human ciliopathies. J. Cell Sci..

[99] Haycraft C.J. (2005). Gli2 and Gli3 localize to cilia and require the intraflagellar transport protein polaris for processing and function. PLoS Genet..

[100] Yoshiba S. (2012). Cilia at the node of mouse embryos sense fluid flow for left-right determination via Pkd2. Science.

[101] Velayos-Baeza A. (2008). The dyslexia-associated gene KIAA0319 encodes highly N- and O-glycosylated plasma membrane and secreted isoforms. Hum. Mol. Genet..

